# The Role of *p16^INK4a^* Pathway in Human Epidermal Stem Cell Self-Renewal, Aging and Cancer

**DOI:** 10.3390/ijms18071591

**Published:** 2017-07-22

**Authors:** Daniela D’Arcangelo, Lavinia Tinaburri, Elena Dellambra

**Affiliations:** 1 Laboratory of Vascular Pathology, Istituto Dermopatico dell'Immacolata, Istituto di Ricovero e Cura a Carattere Scientifico (IDI-IRCCS), Fondazione Luigi Maria Monti (FLMM), via Monti di Creta 104, 00167 Rome, Italy; d.darcangelo@idi.it; 2 Molecular and Cell Biology Laboratory, Istituto Dermopatico dell'Immacolata, Istituto di Ricovero e Cura a Carattere Scientifico (IDI-IRCCS), Fondazione Luigi Maria Monti (FLMM), via Monti di Creta 104, 00167 Rome, Italy; lavinia.tinaburri@gmail.com

**Keywords:** human keratinocytes, *p16^INK4a^*, epidermal stem cells, skin aging, non-melanoma skin cancers

## Abstract

The epidermis is a self-renewing tissue. The balance between proliferation and differentiation processes is tightly regulated to ensure the maintenance of the stem cell (SC) population in the epidermis during life. Aging and cancer may be considered related endpoints of accumulating damages within epidermal self-renewing compartment. *p16^INK4a^* is a potent inhibitor of the G1/S-phase transition of the cell cycle. *p16^INK4a^* governs the processes of SC self-renewal in several tissues and its deregulation may result in aging or tumor development. Keratinocytes are equipped with several epigenetic enzymes and transcription factors that shape the gene expression signatures of different epidermal layers and allow dynamic and coordinated expression changes to finely balance keratinocyte self-renewal and differentiation. These factors converge their activity in the basal layer to repress *p16^INK4a^* expression, protecting cells from senescence, and preserving epidermal homeostasis and regeneration. Several stress stimuli may activate *p16^INK4a^* expression that orchestrates cell cycle exit and senescence response. In the present review, we discuss the role of *p16^INK4a^* regulators in human epidermal SC self-renewal, aging and cancer.

## 1. Introduction

The epidermis is a self-renewing tissue characterized by several compartmentalized layers of keratinocytes in stages of progressive differentiation by virtue of a temporal and spatial gene regulation: Namely, the basal layer is composed by proliferating keratinocytes; the upper layers are composed by viable and differentiated cells; the horny layer is composed by terminally differentiated cells [1]. Epidermal homeostasis relies on keratinocyte stem cells (SCs) hosted in the basal layer; they provide new cells to replace those lost during tissue turnover or following injury. Indeed, SC properties comprise both the long term self-renewal ability and the differentiation capability [1].

## 2. Interfollicular Epidermal Stem Cells

### Two Distinct Models for Interfollicular Epidermis Self-Renewal Proposed in Mice

The hierarchical model suggests the existence of two distinct proliferative cell compartments consisting of slow-cycling SCs and committed progenitor cells. They are hierarchically arranged and contribute to the homeostasis of discrete regions of epidermis. Indeed, slow-cycling cells give rise to actively cycling progenitors that persist for various periods of time in a transient-amplifying (TA) phase and generate differentiated cells [2]. This view is supported by labelling and genetic lineage-tracing experiments displaying an interfollicular epidermis organization into discrete proliferative units (EPU) from the basal to horny layer. During wound healing, mainly SCs contribute to the repair and long-term regeneration [2,3,4].

The stochastic model suggests that the basal epidermis is composed of a single type of population of actively cycling progenitors functionally equivalent, having 50% probability to differentiate or divide. Thus, they directly generate differentiated spinous cells without entering a TA phase [5,6]. Differences in these studies may arise from the tools used to mark the basal cells or from difference in thickness and gene expression in the epidermis at different body sites.

Mouse and human skin have significant differences in cellular architecture and physiology, making it difficult to apply these concepts to human epidermis. Mouse skin has a dense array of hair follicles, whereas human skin displays larger areas of interfollicular epidermis characterized by downward projections of the epidermal rete ridges. Moreover, the human epidermal compartment is thicker with more cell layers than mouse skin. Indeed, human epidermis turnover occurs every 40–56 days, whereas mouse epidermis turns over every 8–10 days [7]. However, genetically marked xenografts of human skin revealed the presence of long-term columns of labeled cells, resembling EPU [8,9]. The first functional demonstration of the presence of human epidermal SCs was achieved in 1975, when they were maintained in culture and propagated for hundreds of generations without losing stemness [10]. Human keratinocyte cultures are constituted of a heterogeneous population of clonogenic cells endowed with different proliferative potential. They can be classified by clonal analysis [11]: The holoclone is generated from SC and has the highest proliferative capacity; meroclones and paraclones originate from young and old TA-cells, respectively. Clonal analysis remains the most reliable means to discriminate human SC from TA-cells. Indeed, none of the identified cell surface proteins is a keratinocyte SC specific marker even though some are able to capture subpopulations containing SCs [11]. Clonal analyses of several human squamous epithelia have unambiguously shown the existence of both self-renewing cells and non-self-renewing TA cells with different clonogenic capacity [12,13]. Both cell types participate in epithelial regeneration [12,14,15]. Thus, the evidence available for human epidermis is consistent with the hypothesis of the presence of both slow-cycling and activated SCs, as found in the mouse. However, the latter SC type may generate TA cells that sustain the longer turnover time and produce differentiated cells, as proposed for other human tissues [16].

For self-renewal, SC has to enter into the cell cycle and divide, and at least one daughter cell must be maintained in an undifferentiated state. Human clonal evolution from SCs to TA cells is a continuous unidirectional process, is instrumental in building up the epidermal structure since TA cells may generate postmitotic terminally differentiated keratinocytes, which migrate upwards and form suprabasal epidermal layers [11]. The SC division timing and the balance of differentiating vs. proliferating cell production are tightly regulated to ensure the SC population maintenance during life. Indeed, whether SCs are exhausted too quickly or their proliferative potential is reduced following endogenous or exogenous damages, tissue atrophy, and premature aging can arise. On the contrary, some environmental stimuli or genomic instability may promote more frequent SC divisions without appropriate differentiation balance that result in abnormal tissue development and even cancer [1].

Several studies indicate the tumor suppressor *p16^INK4a^* as an intrinsic human clonal evolution regulator. Indeed, it increases during in vitro clonal conversion and its forced expression or downregulation impairs this process [17,18,19]. In several tissues *p16^INK4a^* governs the processes of SC self-renewal and its deregulation may result in aging or tumour development [20].

## 3. The *p16^INK4a^* Pathway and Its Regulation

### 3.1. The p16^INK4a^/pRb Pathway

The SC self-renews, taking advantage of the cell cycle machinery to divide. Mitogenic signals induce the expression of D-type cyclins, which bind to and activate cyclin-dependent kinase 4 (CDK4) or CDK6. These complexes inactivate the pRb proteins through phosphorylation that induces pRb-E2Fs dissociation. The E2F transcription factors promote the G1- to S-phase transition through the transcription of their target genes [21].

*p16^INK4a^* is a potent inhibitor of the G1-phase transition of the cell cycle and is considered a tumor suppressor gene. Following several stress stimuli (e.g., DNA damage, oncogenic signals), *p16^INK4a^* directly binds CDK4/6, inhibiting its kinase activity and preventing pRb phosphorylation. Thus, pRb remains associated with E2Fs in the cytoplasm, preventing the E2F-mediated transcription and resulting in cell cycle block [21]. The transition from temporary to stable cell cycle arrest, which involves prolonged cyclin inhibition activity by sustained activation of *p16^INK4a^* (or *p21*) pathway, may be considered the initial step of cellular senescence [20]. *p16^INK4a^* mediated senescence results in chromatin reorganization or senescence-associated heteochromatin foci (SAHFs) which are related to the repression of genes regulated by E2F1 [22].

**Figure 1:**
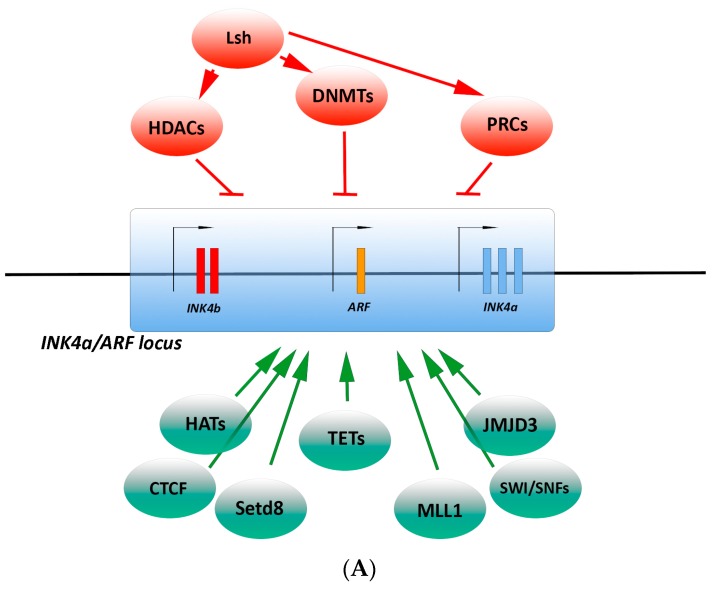

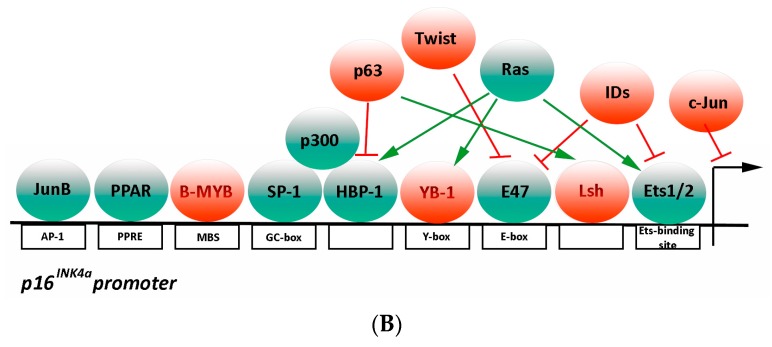
Epigenetic and transcriptional regulation of *p16^INK4a^*. (**A**) Epigenetic regulation of p16^INK4a^—The genomic *INK/ARF* locus is depicted as a bold line, with exons indicated by colored vertical lines (not drawn to scale). The coding regions of *INK4b* (*CDKN2B*) are shown in red, those of *ARF* in orange and those of *INK4a* (*CDKN2A*) in blue. Epigenetic repressors (red) and activators (green) have the opposite function in *INK/ARF* locus regulation; (**B**) Transcriptional activators and repressors of *p16^INK4a^*—The *p16^INK4a^* promoter is depicted as bold line with binding sites indicated by white rectangles (not drawn to scale). Expression of *p16^INK4a^* requires the action of transcription factors (green) that recruit and/or facilitate RNA polymerase association with the promoter. Transcriptional repressors (red) have an opposite function.

The *p16^INK4a^* gene (*CDKN2A*) is contained within the Inhibitor of cdk4/ Alternate Reading Frame (*INK4/ARF*) locus (Figure 1A), a complex locus critical for proper cell cycle control, on human chromosome 9 *p21* [21]. *CDKN2A* encodes two transcripts for *p16^INK4a^* and *p14^ARF^* tumor suppressor genes falling on two distinct reading frames. *p16^INK4a^* is transcribed from exon 1α and exons 2 and 3, whereas *p14^ARF^* is transcribed from exon 1β and exon 2. Different from *p16^INK4a^*, *p14^ARF^* stabilizes and activates *p53*. In addition to *CDKN2A*, the *INK4/ARF* locus encodes a third tumor suppressor, *p15^INK4b^*, which directly blocks the interaction of CDK4/6 with D-type cyclins similarly to *p16^INK4a^*, and the long non-coding transcript *ANRIL* (Anti-sense non-coding RNA in the I*NK4/ARF* Locus), which acts as an epigenetic regulator of the *INK4/ARF* locus [21,23,24,25].

### 3.2. The p16^INK4a^ Expression Regulation

To maintain tissue homeostasis, the ability of *p16^INK4a^* to inhibit cellular proliferation must be tightly controlled. To this aim, the regulation of *p16^INK4a^* expression is complex and involves multiple transcription factors and a finely tuned epigenetic control [24].

#### 3.2.1. Epigenetic Regulation

Epigenetic regulators have specific enzymatic activities, which modify DNA accessibility and chromatin structure, and, in turn, control gene expression (Figure 1A).

DNA methyltransferase (DNMT) and ten-eleven translocation (TET) family enzymes—Conversion of cytosine DNA bases to 5-methyl-cytosine (5mC) is one of the best-characterized epigenetic modifications, which occurs predominantly in CpG islands, and is mostly associated with transcriptional repression. Indeed, 5mC may inhibit transcription by preventing the transcription factor binding to DNA or it may recruit methyl-DNA-binding proteins that facilitate the assembling of chromatin repressor complexes [26]. However, the impact of the DNA methylation on transcription is quite nuanced. Dynamic DNA methylation depends on the interplay between DNMT and TET enzymes. Three DNMT enzymes (DNMT1, DNMT3A, and DNMT3B) catalyze the promoter DNA methylation of several genes codifying proteins able to block cell cycle progression, such as *p16^INK4a^* [27]. Specifically, DNMT1 copies the pattern of methyl marks from the parent strand to the daughter strand after cell division, whereas DNMT3A and DNMT3B catalyze de novo DNA methylation. Although no mechanism of direct demethylation has been identified, TET family enzymes may oxidize 5mC to 5-Hydroxymethylcytosine (5-hmC) that is not recognized by DNMT1 and, in turn, DNA methylation is lost during replication [28,29].

Polycomb group (PcG) protein and Jumanji protein families—In mammals, PcG proteins belong to two classes of complexes, Polycomb Repressor Complex 1 and 2 (PRC1 and PRC2), which operate a transcriptional repression via histone modifications and chromatin compaction. PRC2 functions as an initiator of transcription repression and PRC1 functions as a repressor maintenance complex. These complexes are critical for the homeostatic regulation of the *INK4/ARF* transcription. When they repress the transcription, SCs may enter in the cell cycle [30,31]. The PRC2 complex is composed of methyltransferase subunits Ezh1 and Ezh2, which mediates histone H3 lysine 27 trimethylation (H3K27me3), EED, Suppressor of zeste 12 homolog (Suz12), and Retinoblastoma-associated protein (RBA) p46/48. The H3K27me3 mark mediated by PRC2 is a pre-requisite for PRC1 binding to the chromatin [30]. Multiple variants of canonical PRC1 complex exist and can bind to *INK4/ARF* locus, likely carrying out different biochemical functions. Moreover, the subunit composition is cell type specific [32]. Canonical PRC1 is formed by four different ortholog proteins, CBX (polycomb), PCGF (polycomb group factor), HPH (human polyhomeotic homolog), and the E3-ligase protein (RING) that catalyzes histone H2A monoubiquitylation (H2AK119ub1). This modification prevents RNA polymerase II elongation and promotes chromatin compaction and silencing [32]. PRC1 complexes contain a single representative of the CBX and PCGF subunits. However, these proteins have numerous orthologs, such as five CBX (CBX2, 4, 6, 7, 8), six PCGF (PCGF1, 2, 3, 4, 5, 6), three HPH (HPH1, 2, 3), and two RING proteins (RING1, 2), and can assemble diverse compositions of the PRC1 complex. Different CBX proteins have a distinct pattern of chromatin binding, suggesting that the CBX unit confers specificity to the complex. Indeed, the CBX subunit of canonical PRC1 complex recognizes H3K27me3 via its chromodomain and enables PRC1 recruitment to its target genes [29,30,32]. This results in long-term reversible suppression of *INK4/ARF* genes. The recruitment of PRC1 and PRC2 to the *INK4/ARF* locus is mediated by the long noncoding RNA, *ANRIL*. *ANRIL* binds SUZ12 subunit of PRC2 to induce the Ezh2-mediated H3K27me3 and consequent silencing of the *INK4/ARF* locus. *ANRIL* binds the CBX7 subunit of PRC1, allowing the recognition of H3K27me3 necessary for the H2AK119ub1 and maintenance of silencing. Therefore, *ANRIL* transcription modulation affects the repressing ability of PcG proteins [33,34].

On the contrary, in the presence of stress signals, *p16^INK4a^* expression must be induced to prevent uncontrolled cell cycle progression. The expression of H3K27me3 demethylases of the Jumanji family proteins (JMJD3 and UTX) increases following oncogenic stimuli and removes the repressive histone marks from the *p16^INK4a^* promoter [29]. Jun dimerization protein 2 (JDP2) binds to H3K27, masking it from the actions of PRC2. Thus, JDP2 and JMJD3 work in concert to maintain a permissive state of *p16^INK4a^* expression. In this setting, PRC1 is not able to recognize the unmethylated H3K27, the chromatin in the region surrounding *p16^INK4a^* promoter becomes decondensed and accessible for transcription factor activity [29]. In addition, a cell cycle dysregulation may restrain the PRC2 activity since p53 activation represses EZH2 expression through pRb-mediated inhibition of E2F1 activity [31]. Thus, the homeostatic regulation of INK4/ARF transcription relies on the interplay balance between PRC complexes and histone demethylases.

The histone methyltransferase enzymes—The enzyme SET domain containing (lysine methyltransferase) 8 (Setd8)/lysine methyltransferase 5A (KMT5a) catalyzes mono-methylation of histone H4 at lysine 20 (H4K20me1) that characterizes transcriptionally active genes. Indeed, inhibition of Setd8 induces the reduction of H4K20me1 at several loci, such as INK4a/ARF, and, in turn, increases *p16^INK4a^* expression [35].

The enzyme, histone-lysine N-methyltransferase 2A (KMT2A) or myeloid/lymphoid or mixed-lineage leukemia 1 (MLL1), trimethylates the histone H3 at lysine 4 (H3K4me3), a mark associated with transcriptional activation. MLL1 and the E3 ubiquitin ligase DDB1-CUL4-ROC1 complex directly bind to the *INK4a/ARF* locus and mediate *p16^INK4a^* induction during replicative and oncogene-induced senescence, counteracting PRC-mediated repression [36].

Histone acetyltransferases (HATs) and histone deacetylases (HDACs)—Histone acetylation is a dynamic process controlled by two large families of enzymes with opposed activities: HATs and HDACs. HATs catalyze the acetylation of ε-amino groups of lysine residues within histone tails, facilitating chromatin relaxing and subsequent gene transactivation. The *p16^INK4a^* regulation mediated by the HAT p300 mainly depends on availability of several transcriptional co-factors. Indeed, the transcription factors SP1 and HMG box-containing protein 1 (HBP1), recruit p300 to the *p16^INK4a^* promoter. Both chromatin acetylation and HBP1 foster chromatin decondensation and subsequent gene transactivation [37]. p300 is also able to target the Myb-related protein B (B-Myb) promoter that encodes a *p16^INK4a^* repressor [24]. HDACs remove the acetyl groups and allow a tighter wrap of the histone to DNA that results in gene silencing. All HDACs have been reported to bind and repress transcription from the *p16^INK4a^* promoter. Most of these interactions depend on activity of transcription factors such as Lymphoid Specific Helicase (LSH), HLX1, ZBP-89, and YY1. For instance, HDAC3 and HDAC4 are recruited to *p16^INK4a^* promoter by YY1 to repress its expression. HDAC2 may also directly bind the *p16^INK4a^* promoter [38,39].

Chromatin remodelers—The multisubunit SWI/SNF family complexes contain SWI2/SNF2-like ATPases that hydrolize ATP to disrupt histone-DNA interactions within the nucleosome. This structural alteration results in an open chromatin state suitable for transcription machinery access [40]. As a subunit of the SWI/SNF complex physically interacts with JunB, the SWI/SNF complex may be directly recruited to the *p16^INK4a^* promoter to activate transcription. Moreover, SWI/SNF displaces PcG silencing complexes from *p16^INK4a^* promoter and reduces its DNA methylation [41]. Among SWI/SNF family, BRG/BRM-associated factor (BAF) complexes consist of one ATPase catalytic component (BRG1 or BRM) and several regulatory subunits called BAFs. BRG1 binds to *p16^INK4a^* and *p16^INK4a^*/BRG1 interaction negatively modulates the chromatin remodelling activity of BRG1 itself. However, BRG1 is not required for *p16^INK4a^*-induced cell cycle inhibition. Taken together, these findings indicate a putative feedback loop between *p16^INK4a^* and the SWI/SNF complex [42].

LSH, a member of the SNF2/helicase family, regulates DNA methylation and transcriptional silencing [43]. LSH operates by recruiting DNMTs (Myant and Stancheva, 2008), or through interaction with members of the PRC1 and HDACs [44]. Specifically, LSH represses *p16^INK4a^* expression by recruiting HDAC1 that deacetylates the H3 histone to establish a repressive chromatin structure at the *p16^INK4a^* promoter and delays cell senescence [45].

Nuclear architecture remodelers—The specialized AT-rich binding protein 1 (SATB1) is a genome organizer that interacts with AT-rich sequences and recruits chromatin-remodeling enzymes to regulate chromatin structure and gene expression [46]. Thus, depending on its posttranslational modifications, SATB1 activates or represses multiple genes. SATB1 may interact with pRB/E2F1 complex in gene regulation of *p16^INK4a^* promoter [47].

CCCTC-motif binding factor (CTCF)—CTCF is a zinc finger transcription factor that binds the *INK4/ARF* locus, regulating both chromatin compaction and gene expression. CTCF interaction with the chromosomal boundary is important for *p16^INK4a^* expression maintenance [48]. Indeed, deletion of CTCF resulted in hypermethylation of the *p16^INK4a^* promoter and, in turn, *p16^INK4a^* downregulation. Furthermore, CTCF inhibits epigenetic silencing through chromatin remodeling of the *p16^INK4a^* locus [49,50,51].

#### 3.2.2. Transcriptional Regulation

The presence of several cell cycle genes grouped within the locus *INK4a/ARF* allow an overall regulation by the same chromatin remodelling event(s) following specific stimuli. However, a permissive chromatin state is necessary but not sufficient for gene expression. Indeed, transcription requires the binding of activation factors to specific enhancer and promoter elements and the subsequent recruitment of RNA polymerase. Transcription factors model the precise gene expression pattern required for specific functions, binding cooperatively to DNA in the form of cell-type-specific multiprotein complexes [24,25]. Transcriptional regulation of the *p16^INK4a^* gene is finely controlled by several transcription factor antagonistic pathways (Figure 1B).

Ets-binding site-mediated regulation—The E-box binding transcription factors E-26 transformation-specific (ETS) proteins (Ets1 and Ets2) are activated by the RAS-MAP kinase pathway following dangerous stimuli. Ets 1 and Ets 2 bind to *p16^INK4a^* gene and increase its transcription, leading to cellular senescence in fibroblasts [52]. To suppress *p16^INK4a^* overexpression, the Inhibitor of DNA Binding 1 (ID1), a helix loop helix (HLH) transcription factor, binds Ets transcription factors and inhibits their activity [52]. Thus, the balance between Ets1/2 and ID1 seems to act as a sensor of aberrant growth signals.

E-box-mediated regulation—E47 is a basic helix-loop-helix (bHLH) protein that homo- and heterodimerizes with other HLH proteins and binds two consensus sequences in the E-box element to activate *p16^INK4a^* expression. ID1 may influence the transactivation activity of E47 acting as dominant negative following heterodimerization. TWIST1 inhibits *p16^INK4a^* transcription by decreasing E47 expression [53]. Myc is an E-box-binding transcription factor that binds the *p16^INK4a^* promoter to upregulate its expression in human cells [54].

Y box-mediated regulation—Y box-binding protein 1 (YB-1) is a transcription factor that represses the *p16^INK4a^* transcription through direct association with its promoter region, resulting in the cell growth promotion and cellular senescence prevention [55].

Sp1-binding site-mediated regulation—The GC-rich region within the *p16^INK4a^* promoter contains at least five putative GC boxes that represent the putative binding target sites for Sp transcription factors [53,56,57]. A positive transcription regulatory element in the *p16^INK4a^* promoter harbors a GC box for Sp1 binding [56] that is enhanced during cellular senescence mainly due to an increase in Sp1 binding affinity. Sp1 positively regulates *p16^INK4a^* transcription by direct binding to DNA and recruiting P300 to the *p16^INK4a^* promoter [57].

HBP1-binding site-mediated regulation—*p16^INK4a^* promoter contains a HBP1 binding site. This is a downstream effector in the Ras/MAPK signaling pathway positively regulating *p16^INK4a^* transcription by direct binding to DNA and recruiting P300 to its promoter [37]. Of note, HBP1 also represses the DNMT1 gene, resulting in both *p16^INK4a^*-specific and global DNA hypomethylation changes [58].

INK4a transcription silence element (ITSE)-mediated regulation—The *p16^INK4a^* promoter harbors a negative regulatory element that contains a binding site for B-Myb, a transcription factor involved in the regulation of cell survival, proliferation, and differentiation [59].

Ap1 site-mediated regulation—AP1 proteins are homodimers and heterodimers composed of basic region-leucine zipper (bZIP) proteins, including Jun proteins (c-Jun, JunB, JunD), Fos proteins (c-Fos, FosB, Fra-1 and Fra-2), Jun dimerization partners (JDP1 and JDP2), and the closely related activating transcription factors (ATF2, LRF1/ATF3, and B-ATF). JunB activates *p16^INK4a^* transcription by binding to three identified AP1-like sites within the *p16^INK4a^* promoter. Conversely, c-Jun acts as a JunB antagonist, downregulating *p16^INK4a^* expression. Interestingly, *p16^INK4a^* is able to bind to JNKs and inhibit c-Jun phosphorylation and AP-1 activity [24,60].

Peroxisome proliferator response element (PPRE)—Peroxisome proliferator-activated receptor alpha (PPARα) specifically binds to the canonical PPRE region of the *p16^INK4a^* promoter and interacts with Sp1, enhancing *p16^INK4a^* expression. PPARγ phosphorylation represses its transactivation function [61].

## 4. *p16^INK4a^* and Epidermal Homeostasis

A key requirement for SC maintenance throughout life is the *p16^INK4a^* repression [20]. Indeed, its increase may be considered a barrier to SC pluripotency. For instance, during reprogramming of induced pluripotent SCs (iPS), the silencing of *INK4/ARF* gene transcription is associated with SC marker induction [25].

### 4.1. The p16^INK4a^/pRb Pathway in Epidermal Homeostasis

*p16^INK4a^* expression is undetectable during embryogenesis and in young tissues. In human epidermis, *p16^INK4a^* is undetectable in primary human keratinocyte cultures from young donors [17,18,19]. *p16^INK4a^* is a key regulator of clonal conversion and its inactivation in epidermal SCs is necessary for maintaining their stemness [18]. When *p16^INK4a^* is repressed, CDK4-cyclin D1complex is in the active state and allows E2F-mediated transcription of gene involved in cell cycle progression through inhibition of pRb. Indeed, the CDK4-cyclin D1complex regulates keratinocyte proliferation, but is not implicated in calcium-induced keratinocyte differentiation [62,63,64]. Specifically, E2F1 maintains primary human keratinocytes in a proliferative and undifferentiated state. During differentiation, E2F1 levels decrease. Moreover, E2Fs become upregulated after wounding. Specifically, E2F1 and E2F2 are expressed in all layers of proliferating keratinocytes and in migrating cells at the wound margin [63]. Activation of E2F transcription results in increased transcription of DNMT1 that, in turn, methylates *p16^INK4a^* promoter [23].

*INK4/ARF* locus activation plays a limited role in skin development and homeostasis [65]. However, deregulation of epigenetic pathways regulating epidermal homeostasis, induces *p16^INK4a^* expression [66,67,68,69,70,71]. Thus, *p16^INK4a^* may be considered a master sensor of aberrant chromatin status that rapidly drives cell cycle exit, inhibiting CDK4-cyclin D1 complex activity [72]. The pRb and E2F family members modulate genes involved either in cell cycle exit and differentiation. The three pRb family proteins are expressed in human epidermis. Specifically, pRb and p107 proteins are expressed in all layers, whereas p130 is restricted to suprabasal keratinocytes, suggesting a different coordinated role during epidermal differentiation. pRb in conjunction with p107 plays a central role in regulating epidermal homeostasis. pRb is essential for the maintenance of the terminally differentiated state, preventing cell cycle re-entry, and p107 compensates for the effects of Rb loss [73]. Rb proteins modulate the epidermal homeostasis by differential interaction with E2Fs. Indeed, E2F family members are divided into two groups: activators (E2F1-E2F3a) and repressors (E2F3b-E2F8). E2F1 and E2F4 appeared to exert opposite functions in cell differentiation [74]. Beyond its role as cell cycle repressor, E2F4 regulates expression of several genes involved in cell fate decisions [63,75,76]. To regulate differentiation, apoptosis, or senescence, pRb-E2F complexes can also recruit co-repressors/activators (e.g., HDAC1, HDAC2, DNMT1, HATs, BRG1) to regulate chromatin structure and transcription [76].

### 4.2. The p16^INK4^ Epigenetic Modulators and Transcription Factors in Epidermal Homeostasis

**Figure 2:**
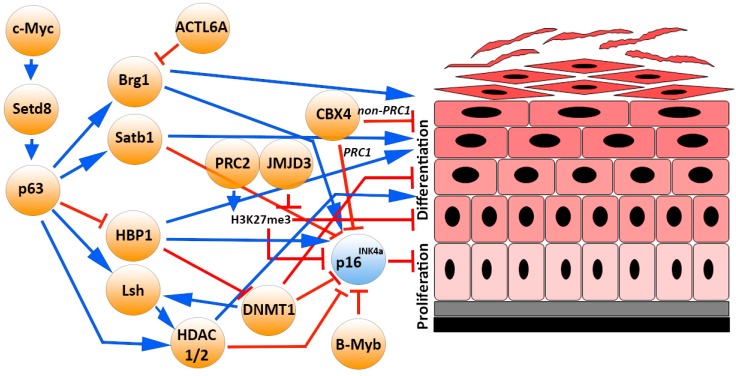
Epigenetic regulation of *p16^INK4a^* in epidermal homeostasis. The interfollicular epidermis is a stratified epithelium in which cell proliferation and differentiation are compartmentalized and tightly regulated. Cell proliferation occurs in the basal layer. When keratinocytes withdraw from cell cycle, they generate post-mitotic cells, which migrate upwards and form suprabasal layers executing their terminal differentiation program. Epigenetic modifiers (orange circles) that regulates *p16^INK4a^* (blue circle) expression and/or keratinocyte differentiation in epidermal homeostasis are indicated. Blue arrows and red lines indicate positive or negative actions, respectively.

Multiple *p16^INK4a^* epigenetic regulators (described in paragraph 3) cooperatively modulate the balancing between epidermal SC maintenance and differentiation (Figure 2). Specifically, some epigenetic enzymes may preserve stemness and promote proliferation by repressing *p16^INK4a^* itself and other cell-cycle inhibitor transcription, as well as inhibiting unscheduled activation of non-lineage- or terminal differentiation-associated genes (e.g., DNMT1, HDAC1/2, PCR1, and PCR2 subunits). On the contrary, the *p16^INK4a^* transcription activators may also promote keratinocyte terminal differentiation acting on epidermal differentiation complex (EDC) genes (e.g., JMJD3, BRG1, SATB1) [28,29,38,77,78,79].

DNA methylation is relevant for tissue homeostasis as it provides functional variability to maintain the equilibrium between SC proliferation and differentiation. In skin SCs, the differentiation gene promoters are methylated and lose this repressive mark following cell fate commitment. Indeed, DNMT1 is mainly expressed in the epidermal basal layer, and decreases with keratinocyte differentiation [66,80]. Conditional ablation of DNMT1 in mouse epidermis results in marked de-repression of *p16^INK4a^* and differentiation genes. Epidermis becomes thickened, likely due to aberrant basal cell differentiation [80]. The lack of DNMT1 in human epidermal progenitor cells leads to abnormal induction of cell cycle regulator genes, such as *p16^INK4a^*, and loss of the tissue self-renewal capacity. However, depletion of DNMT1 in human keratinocytes is not sufficient to induce the loss of cell identity. Thus, DNMT1 displays a critical role in maintaining epidermal progenitor cells and tissue renewal [66]. Genes associated with cell proliferation and self-renewal are repressed by de novo methylation in differentiated cells [81]. Of note, 5-hmC is very low in SCs and differentiated keratinocytes, suggesting that TET enzymes might not play a key role in skin homeostasis [82].

The transcriptional repressor PcG proteins are essential to control SC identity and proliferation by preventing unscheduled induction of non-lineage and differentiation genes. Indeed, in keratinocyte SCs, non-epidermal and differentiation genes are H3K27me3-repressed whereas this repressive mark is lost during cell differentiation. In committed cells, stemness genes display high levels of H3K27me3 mark [29]. The role of the PRC2 complex in skin development and homeostasis has been investigated by loss-of-function studies. Knocking out both Ezh1/2 subunits in embryonic epidermal progenitors is necessary for the complete ablation of PRC2 complex activity and subsequent loss of the H3K27me3 mark. PRC-mediated repression maintains progenitor cells by repressing both suprabasal and Merkel cell lineages. Specifically, it prevents binding of AP-1 to its target genes, such as EDC genes, and represses the SOX2 gene required for Merkel cell specification. Moreover, PRC-mediated repression plays a key role in skin homeostasis: Ezh1/2 loss triggers the activation of *INK4a/ARF* locus, impairing proliferation and hair follicle development [68,69,83]. Loss of function of EED or Suz12 has similar skin phenotypes to Ezh1/2 loss. Thus, in the epidermis EED, Suz12 and Ezh1/2 function mainly as subunits of the PRC2 complex, although these subunits may act independently of the PRC2 complex [84]. Jarid2, a PRC2 ancillary protein, is necessary to maintain scheduled proliferation in epidermal homeostasis. Jarid2 loss leads to cell proliferation reduction and differentiation increase in progenitors, without affecting epidermal development, and hair growth delay [85].

PRC1 functions have been investigated in human keratinocytes and the most studied proteins are the PCGF4 subunit/Bmi-1 and Cbx4. Bmi-1-mediated regulation of *p16^INK4a^* expression is required for proper development, SC maintenance, and homeostasis in several tissues [86]. However, Bmi-1 is expressed in several epidermal layers, although a decrease expression gradient exists from basal to upper layers [87,88,89,90]. Of note, Bmi-1 is localized in the nucleus of proliferating keratinocytes in culture [18,90,91] and in the cytoplasm of senescent keratinocytes [18,91], suggesting that irreversible growth arrest and terminal differentiation are associated with a decrease of Bmi-1 expression and cytoplasmic distribution. Bmi-1 promotes human keratinocyte survival preventing apoptosis [90]. Bmi-1 and other PcG protein expression inversely correlate with keratinocyte clonogenic and proliferative potential even though these proteins cannot be strictly considered SC-specific proteins in the epidermis [19,69,90]. Human epidermal SC moves from a slow-cycling to an actively-proliferating status to ensure normal homeostasis. Although these states differ in their cell cycle profile, in both cases differentiation and senescence processes have to be blocked. Cbx4 uniquely possesses, among PRC1-associated Cbx subunits, both PRC1 chromodomain- and Small Ubiquitin-like Modifier (SUMO) E3 ligase-dependent activities [92]. Cbx4 ablation in mice demonstrates that it maintains epithelial identity by repressing non-epidermal lineage genes and inhibits senescence by repressing the *INK4a/ARF* locus in epidermal progenitor cells through its canonical PRC1 activity. On the other hand, it controls both proliferation of basal keratinocytes and inhibition of their premature differentiation by its SUMO E3 ligase activity [93]. In human epidermal SCs, Cbx4 displays an anti-senescent function by its PRC1 activity whereas inhibits SC activation and differentiation through its SUMO E3ligase activity. These findings provide evidence that differentiation and senescence are independent processes in human epidermis. Specifically, Cbx4 promotes a slow-cycling state of SCs, preventing their differentiation and senescence through repression of Bmi-1, DNMT1, and Ezh2 and, in turn, the transition to the active SC state [70]. The removal of H3K27me mark during cell differentiation is catalyzed by JMJD3. Indeed, human keratinocytes expressing JMJD3 exhibit premature differentiation whereas its loss impairs differentiation [67].

HDAC1 and HDAC2 play a critical role in the gene expression control of the epidermal progenitor cells and epidermal differentiation. They are expressed in all epidermal layers but display a higher nuclear expression level in terminally differentiated cells [71]. Conditional ablation of either HDAC1 or HDAC2 does not disrupt epidermal homeostasis [94]. However, a double knock-out HDAC1/2 mouse model displays that deficient epidermis upregulates p21 and *p16^INK4a^*, fails to differentiate and remains in single-layer, similarly to p63-null mice. Thus, HDAC1/2 mediates repressive functions of p63 in epidermal development, and suppress p53 activity [71]. Moreover, SCs of interfollicular epidermis and hair follicle exhibit hypoacethylated histone H4. When cells exit from the quiescent state to proliferate and differentiate, they display an increased acetylation level [95]. Of note, the histone deacetylase inhibitor Trichostatin A (TSA) induces premature senescence in primary human keratinocytes with concomitant Bmi-1 and Ets-1 reduction and *p16^INK4a^* increase [19], as well as terminal differentiation [96].

Altogether, these data indicate that DNMT1, PRC1, PRC2, and HDAC proteins work in concert to protect epidermal cells from senescence by repressing *p16^INK4a^* expression and preventing differentiation. Furthermore, balancing these enzymatic activities regulates the transition between epidermal SC quiescence and activation.

These epigenetic mechanisms may be regulated by specific transcription factors, such as p63, which is considered a master regulator of epidermal morphogenesis and SC maintenance [97,98]. It is regulated by the chromatin remodeler Setd8 and has an important role in transcription and higher-order chromatin remodeling through its direct targets—HBP1, LSH, SATB1, and BRG1—which also modulate *p16^INK4a^* expression. Setd8 is a Myc target and is weakly expressed in skin. Deletion of Setd8 during embryogenesis results in *p63* loss and a failure to form the epidermis. Setd8 activity is mainly present in dividing basal progenitor cells and is required for normal tissue homeostasis [35]. Indeed, inducible and conditional ablation of Setd8 in the epidermal basal layer results in cell-cycle arrest, apoptosis of progenitor cells and disruption of skin homeostasis. Thus, Setd8 is required for epidermal progenitor cell survival by inducing p63 and repressing p53 levels [99]. In the epidermal basal layer, *p63* directly represses HBP1 and, in turn, *p16^INK4a^* expression. HBP1 is also required for differentiation and stratification processes [100]. Moreover, the chromatin remodeler LSH is a direct target of both p63 [101] and DNMT1 in primary keratinocytes. Thus, p63 and DNMT1 may cooperate to repress *p16^INK4a^* transcription and maintain basal progenitor proliferation. The p63-target SATB1 plays a key role in epidermal morphogenesis, controlling tissue-specific chromatin remodeling and *p16^INK4a^* repression in epidermal progenitor cells [102,103]. *p63* directly regulates the expression of the SWI/SNF complex BRG1 that displays a critical role in skin homeostasis. It remodels the higher-order chromatin structure of the EDC for the efficient expression of differentiation genes in epidermal progenitor cells [104]. BRG1 is expressed in all epidermal layers but its activity is restricted to suprabasal layers and BRG1 deficiency leads to defects in terminal differentiation accompanied by the formation of non-functional epidermal barrier [104,105]. Indeed, the ACTL6a subunit, which binds the SWI/SNF complex, is differentially expressed and regulates epidermal differentiation [106]. The remodeler CTFC is mainly expressed in the nuclei of suprabasal keratinocytes and its expression diminishes in the more differentiated layers of the epidermis according to its function to limit cell growth [107].

Several *p16^INK4a^* promoter transcription factor regulators (described in paragraph 3) may have a role in epidermal proliferation and differentiation. Ets1 is expressed in the epidermal basal layer, blocks keratinocyte terminal differentiation, and induces expression of matrix metalloproteases and innate immune mediators [108]. Ets1 transgene induction in basal layer of mouse epidermis results in tissue hyperplasia and impaired differentiation. Indeed, Ets1 disrupts Notch signalling in part through *p63* upregulation [109]. Ets2 is highly expressed in differentiated keratinocytes. The *p16^INK4a^* repressor Id-1, Id-2, and Id-3 proteins are expressed in proliferating human keratinocytes and downregulated in differentiated cells. Of note, a cytoplasmic Id-1 expression and nuclear Id-2 and Id-3 expression has been observed in the epidermal basal layer [110]. The *p16^INK4a^*-negative regulator B-Myb plays an important role in maintaining the undifferentiated phenotype of keratinocytes in the basal epidermal layer [111]. The ubiquitously expressed Sp1 and AP-1 (c-Jun, JunB, JunD, c-fos, FosB, Fra-1, and Fra-2) transcription factors are expressed at different levels in multiple epidermal layers and regulate competing processes in the epidermis (proliferation, survival, and differentiation) to maintain physiological homeostasis [112]. Of note, Ezh2 controls the proliferative potential of epidermal progenitors by repressing the *INK4A/ARF* locus and the terminal differentiation by preventing the recruitment of AP-1 to promoters of genes governing barrier formation [69]. Specifically, Fra-2 is methylated on lysine residues by Ezh2 in basal keratinocytes to repress EDC genes [113]. PPARα is an important regulator of epidermal development and homeostasis as it is involved in controlling the switch from proliferating basal to differentiating suprabasal keratinocytes [114].

## 5. *p16^INK4a^* and Epidermal Aging

Aging is a complex process characterized by accumulation of macromolecular damage and compromised tissue renewal. Such decrease of tissue regenerative capacity is not necessarily due to a SC-number or self-renewal decline but rather to a reduced ability to produce progenitors and, in turn, differentiated effector cells [115]. Epidermal mouse SCs isolated from young and old mice do not display substantial difference in number and gene expression pattern [116,117,118]. Therefore, the epidermis is able to maintain the barrier properties for the entire organismal life-span although the tissue regenerates and heals more slowly. Of note, epidermal grafts from older individuals allow a permanent coverage of burnt skin [12,119]. Experiments in old mice show that the slow regeneration rate is likely due to changes in TA-cell kinetics following damage accumulation. This leads to a decrease in the cellular output of TA cells and a decrease in the rate of TA cell proliferation. However, the number of TA cells increases in aged epidermis likely as they progress more slowly during the cell cycle than young TA cells [117,120].

One aging hallmark is cellular senescence since the number of senescent cells increases in tissues during organismal lifespan. Cellular senescence is triggered by several different intrinsic and extrinsic stimuli that induce growth arrest and distinctive phenotypic alterations, including considerable chromatin and secretome changes. In young organisms, cellular senescence prevents the uncontrolled proliferation of damaged cells. However, in old organisms the continuous and cumulative damages and the deficient clearance of senescent cells results in their accumulation with deleterious effects on tissue homeostasis [121,122,123].

### 5.1. The p16^INK4a^/pRb Pathway in Epidermal Aging

*p16^INK4a^* has a well-established role in mediating and maintaining cellular senescence during both replicative and stress-induced senescence processes. Thus, it may be considered an effector of aging [122,123]. Indeed, the expression of *p16^INK4a^* is undetectable in unstressed and healthy tissues of young mammals [124,125]. *p16^INK4a^* accumulates during tissue aging and therefore is considered one of the most robust aging biomarkers characterized to date [124,125,126,127]. Specifically, data from progeroid and calorically restricted rodents suggest that *p16^INK4a^* may be a marker of biological rather than chronological aging [125,128]. Similarly, smoking and chemotherapy are associated with elevated *p16^INK4a^* expression in humans [129,130]. Assessing its effect on aging has been difficult since *p16^INK4a^*-null mice are tumor-prone [65]. However, in mouse models with conditional expression of *p16^INK4a^* it causes a decline of regenerative capacity of hematopoietic, neural SC and intestinal cells of young adult mice, rapidly inducing several features of premature aging. De-induction of *p16^INK4a^* revealed that these features are strikingly reversible [124,127,131]. Inducible elimination of *p16^INK4a^*-positive senescent cells in a progeroid mouse model delays the onset of premature aging phenotypes in different tissues, such as adipose tissue, skeletal muscle, and eyes; attenuates the progression of already present age-related disorders; and extends healthspan [128].

*p16^INK4a^* expression directly correlates with chronological aging of human skin in vivo. The number of *p16^INK4a^* positive cells increases with age in both epidermal and dermal compartments [87]. Furthermore, the number of *p16^INK4a^* positive cells in human skin is also a marker of biological age in middle-aged individuals with longevity propensity compared to their age-matched partners [132]. A strong positive correlation between paraclone increase and *p16^INK4a^* expression at first culture passage has been observed in human keratinocyte from elderly donors [19]. *p16^INK4a^* levels increase in the epidermal basal layer in the aged model of skin equivalent in concomitance with a proliferation marker decrease. In genetically-modified skin equivalents, *p16^INK4a^* overexpression in cultures from young donors results in a decrease of proliferation markers whereas *p16^INK4a^* silencing results in morphology improvement and proliferation markers restoration in cultures from old donors [18,133].

### 5.2. The p16^INK4^ Epigenetic Modulators and Transcription Factors in Epidermal Aging

An additional common age marker is genomic and epigenomic instability due to damage accumulation throughout life. Alterations in DNA methylation or histone acetylation/methylation, as well as other chromatin-associated protein modification, can induce epigenetic changes [121]. Thus, epidermal aging may depend on the impairment of epigenetic factor activity on *p16^INK4a^* promoter regulation that represses the *INK4a/ARF* locus. Indeed, oxidative stress induces demethylation of *p16^INK4a^* promoter and, in turn, human keratinocyte senescence [134]. The expression of PcG proteins declines in aged epidermis and their decrease is associated with keratinocyte senescence both in vivo and in cell culture models [19,87]. PRC2 expression gradually decreases with the age of basal epidermal progenitors. Ezh2 decrease leads to reduction of H3K27 trimethylation levels, disassociation of PRC1 from the chromatin and increased transcription of *INK4a/ARF* genes [78]. Indeed, early expression of *p16^INK4a^* in human keratinocytes from old donors strongly correlates with Bmi-1 downregulation. What is more, Bmi-1 modulates both *p16^INK4a^* levels and clonal conversion in primary human keratinocytes from elderly donors. Specifically, Bmi-1 overexpression inhibits *p16^INK4a^*-promoter activity and decreases the protein expression. These Bmi-1 overexpressing keratinocyte cultures display a delay of cell senescence and a restoration of keratinocyte clonal ability, showing a phenotype similar to that observed in age-matched healthy control subjects [19]. Of note, pRb seems necessary for Bmi-1 function in the transcription repression of *p16^INK4a^*, since the deletion of pRb from cells resulted in histone H3K27 trimethylation loss that impairs the Bmi-1 recruitment to the *p16^INK4a^* locus [135]. The enzyme MLL1 is repressed by PRC complexes during proliferation of young cells and mediates *p16^INK4a^* induction during replicative and premature senescence [36]. The chromatin remodeler Setd8 is downregulated in both oncogene-induced and replicative senescence and inhibition of Setd8 alone is sufficient to trigger senescence [35]. Deficiency of its target, *p63*, induces cellular senescence in human proliferating keratinocytes [136] and leads to accelerated skin aging in *p63* heterozygous mice [137]. Also, the *p63*-targets LSH and SATB1 play a key role in senescence and aging. Indeed, LSH-deficient mice exhibit accelerated aging and enhanced cellular senescence [138]. E2F1 regulates LSH transcription, therefore its repression during senescence decreases LSH expression [139]. SATB1 expression decreases during human keratinocyte replicative senescence and its silencing is able to induce a G1 cell cycle arrest concomitant with an increase of senescence-associated markers, such as *16^INK4a^* [103]. Of note, *16^INK4a^* and the pRB/E2F pathway are critical for SATB1-induced cell cycle arrest. Indeed, SATB1 causes anchorage-independent growth and invasive phenotype in *16^INK4a^* deficient cells [47]. The chromatin remodeler BRG1 may drive SAHF formation via its chromatin remodeling activity and/or by upregulating *p16^INK4a^* expression. During senescence BRG1 is overexpressed and interacts with pRB to drive SAHF formation. On the other hand, this interaction is dispensable for upregulating *p16^INK4a^* and *p21* by BRG1, which depends upon its chromatin remodeling activity [42]. Interestingly, age-related metabolic pathologies are associated with PPARs activation that induces the *16^INK4a^* expression and leads to subsequent cell cycle arrest and senescence [61].

Following acute stress stimuli, the Ras/MAPK pathway induces Ets1 and HBP1 upregulation that triggers premature senescence via *16^INK4a^* transcription [52,140]. The age-related increase of *16^INK4a^* expression in mouse and rat tissues has been attributed mainly to Ets-1 expression [125]. Moreover, the marked increase in *16^INK4a^* levels observed in senescent human fibroblasts is consistent with the reciprocal reduction of Id-1 and accumulation of Ets-1 [52]. However, although Id-1 overexpression is able to extend the keratinocytes’ lifespan, it does not prevent the onset of replicative senescence [141,142]. Despite the ability of Ets-1 and Id-1 to modulate the *16^INK4a^* promoter in primary human keratinocytes, no correlation between their expression and 16*^INK4a^* expression or SC depletion during skin chronological aging has been detected [19]. Ras/MAPK pathway also promotes senescence through the activation of JMJD3 and down regulation of Ezh2 that, in turn, cooperatively activates *16^INK4a^* transcription [143]. Moreover, Ras pathway induces ROS increase that inhibits DNMT1 expression through E2F inhibition. Thus, reduced methylation activity may derepress the 16*^INK4a^* promoter [23]. The *16^INK4a^*-repressor B-Myb is regulated by cyclin D1. Thus, its decrease following stress may regulates entry into senescence [144]. During replicative senescence and upon RAS overexpression, *ANRIL* expression is suppressed. Thus, cell-cycle inhibitors *p14^ARF^*, *p15^INK4b^*, and *16^INK4a^* are upregulated, likely by reduced interaction with PRC complexes, and senescence is triggered [23].

## 6. *p16^INK4a^* and Non-Melanoma Skin Cancers

Non-melanoma skin cancers (NMSCs) are the most frequent cancers in the Caucasian population. NMSCs, mainly squamous-cell carcinomas (SCCs) and basal-cell carcinomas (BCCs), originate from keratinocytes and are the most common age-associated malignancies [145,146]. The exponential increase of tumor frequency with age may be in part due to the presence of senescent cells next to preneoplastic cells. Senescent cells accumulate in skin and secrete several enzymes, which disrupt the cellular microenvironment, and many inflammatory cytokines and growth factors that can stimulate tumor cell growth. Thus, relatively few senescent cells might compromise skin function and integrity [123,147].

Cutaneous SCCs (cSCCs), especially less well-differentiated tumors, have metastatic potential. They represent an interfollicular epidermis neoplasia and may originate from premalignant actinic keratoses (AKs) and “in situ” SCC (ISSCCs). The most important alterations in cSCC etiology involve RAS, *p53* and *16^INK4a^*/pRb pathways [148]. BCCs rarely metastasize but are locally invasive and can be disfiguring. BCCs likely arise from hair follicle SCs and their etiology is highly dependent on Hedgehog signaling pathway dysregulation [149]. However, a coupled role of *16^INK4a^* and Hedgehog signaling pathway is observed in the BCC pathogenesis [150].

Cells with dysfunctional *16^INK4a^* display uncontrolled proliferation bypassing G1/S transition checkpoint. The *16^INK4a^* gene is frequently mutated in human cancers [20] and mice lacking *16^INK4a^* are prone to spontaneous or induced tumorigenesis [151,152]. However, studies performed on NMSCs led to controversial results about *16^INK4a^* expression. Some reports correlated higher malignancy with *16^INK4a^* loss [153,154] and others with *16^INK4a^* overexpression [155,156,157,158,159]. Numerous mutations of 16*^INK4a^* have been found in AKs and cSCCs, such as homozygous gene deletions, hypermethylation of its promoter, and mutations in the *INK4a/ARF* locus. Loss of heterozygosity in at least one locus of the 9p21 region (containing the CDKN2A gene) is observed in 20% of AKs and 46% of cSCCs, suggesting that *16^INK4a^* inactivation may have a critical role in skin lesion progression [160]. *16^INK4a^* gene may be mutated in up to 24% of cSCCs and these mutations may be hereditary or acquired following UV-induced damage [156]. In BCC, *16^INK4a^* may also be inactivated apart from loss of heterozygosity, promoter hypermethylation, and/or mutations in the *INK4a/ARF* locus [161]. On the contrary, several immunohistochemical studies report an increased *16^INK4a^* expression in ISSCCs compared to AKs and in poor differentiated cSCC compared to well differentiated [157,159,162,163,164]. In premalignant AKs, *16^INK4a^* expression correlates with a functional pRb pathway, suggesting that upregulation is the result of senescence induction following stress stimuli [165]. Human papillomavirus (HPV) is well established as a risk factor for oropharynx SCCs. Neverthless, improved survival is observed in patients with HPV-positive SCCs [166,167]. HPV oncoprotein E7 directly inactivates pRb [168], thus 16*^INK4a^* overexpression may be a compensatory feedback effect of the inability to control cell proliferation and has been suggested as marker for HPV-associated SCCs [169]. However, a subgroup of aggressive cSCCs displays high *16^INK4a^* expression in the absence of HPV [165,170]. *p16^INK4a^* upregulation might occur as a response to pRb pathway dysfunction. Indeed, *16^INK4a^* expression correlates with the lack of pRb phosphorylation in ISSCCs and invasive cSCCs [165]. Alteration in the pRb pathway can also induce an aberrant cytoplasmic localization of *p16^INK4a^* due to CDK4 sequestration [171]. Alternatively, the protein might be present in a mutated but inactive form [161,172]. Moreover, a strong *16^INK4a^* expression is observed in BCC [173]. In highly invasive cSCC and BCC subtypes, *p16^INK4a^* is overexpressed at the infiltrative front followed by ceased proliferation, suggesting that *p16^INK4a^* could be involved in tumor invasion independent by proliferation effects. Indeed, *16^INK4a^* is implicated in the regulation of matrix-dependent cell migration [165,174]. However, although the expression of *p16^INK4a^* and Ki67 is associated with both BCCs and cSCCs, their expression does not seem to correlate with the degree of proliferation and malignancy. Instead, *p16^INK4a^* overexpression is significantly associated with sun-exposed areas, suggesting a possible induction of *p16^INK4a^* overexpression by UV radiation [172]. Interestingly, UV exposure induces DNMT expression in the epidermis [175], suggesting that mainly histone modification might be the cause of *p16^INK4a^* expression.

### 6.1. Oncomine Analysis

Further analyses concerning expression of epigenetic modulators and transcription factors (described in paragraph 3) are needed to study *p16^INK4a^* expression in-depth in NMSCs, independently from HPV infection. Here, we examined the expression of 50 genes, including *p16*^INK4^, downstream pRb pathway proteins and *p16^INK4^* promoter modulators, as reported in human skin cancer datasets available at Oncomine (www.oncomine.org) as previously described [176,177]. Two independent datasets (Nindl skin and Riker melanoma) [178,179] from epidermal cancers (AKs, cSCCs and BCCs) were investigated as indicated in details in Table 1 and Table 2. Gene expression in tissue biopsies from 45 patients was analyzed, namely, 10 control normal tissues and 35 cancer samples. Moreover, a comparative analysis on datasets from other epithelial cancers (75 samples from head and neck SCCs (HNSCCs) and 73 samples from oral cavity SCCs (oSCCs) vs. 43 normal samples) has been performed. Four independent datasets (Ginos Head-Neck, Cromer Head-Neck, Peng Head-Neck and Toruner Head-Neck) [180,181,182,183] from HNSCCs and from oSCCs were investigated as indicated in details in Table 1 and Table 3. Gene expression was evaluated by setting “Cancer vs. Normal analysis” and choosing as Cancer Type: “Actinic (Solar) Keratosis”, “Skin Squamous Cell Carcinoma”, “Skin Basal Cell Carcinoma”, “Head and Neck Squamous Cell Carcinoma”, and “Oral Squamous Cell Carcinoma”. Expression fold change (Cancer vs. Normal samples) and p values are reported for each analysis. We mainly focus the discussion on expression values showing fold changes >2 and statistical significance (*p* < 0.01).

#### 6.1.1. *p16^INK4^* Downregulation in Epithelial Tumors

A *p16^INK4^* decrease is observed in one dataset from HNSCCs (Cromer dataset—Table 3). Accordingly, the cell cycle regulators CDK4 and Cyclin D1 and the pRb pathway effectors E2F1 and E2F4 are significantly upregulated in these samples. Cyclin D1 upregulation may also be a sign of Ras activation [184]. Of note, the expression of some *p16*^INK4^-regulators (Ets1/2, YB1, AP-1 proteins), which are downstream targets of the Ras pathway, is significantly increased (Cromer dataset—Table 3), suggesting that dysregulation of pRb and Ras pathways might work in concert to sustain tumor progression in these samples.

#### 6.1.2. *p16^INK4^* Upregulation in Epithelial Tumors

A *p16^INK4^* increase is observed in NMSCs, HNSCCs (Ginos data set) and oral cavity SCCs (oSCCs) (Table 2 and Table 3). Information regarding patient HPV-positivity is not available from these datasets. In the Nindl dataset, skin biopsies are obtained from organ-transplanted patients with multiple NMSC lesions mainly located on sun-exposed areas. Of note, immunosuppressed patients display an increased HPV prevalence in cSCC compared to immunocompetent patients [185].

##### The *p16^INK4a^*/pRb Pathway in Epithelial Tumors

Different from AK samples, cSCCs, and BCCs display a significant increase of CDK4 expression as HNSCCs and oSCCs samples (Table 2 and Table 3). CDK4 is overexpressed in human cSCCs and is sufficient to induce epidermal tumorigenesis concomitant with oncogenic Ras expression [184]. In AKs and cSCCs, Cyclin D1 slightly increases (Table 2) as previously described [162]. Manojlovic-Gacic and colleagues show that Cyclin D1 is more expressed in ISSCC compared to AKs and in poor-differentiated cSCC compared to well-differentiated cSCC. Thus, the increase of its distribution through pre-invasive phases in cSCC development may reflect its role in the skin-lesion progression. Of note, the Cyclin D1 expression distribution correlates with that of *16^INK4a^* [162]. In poor differentiated HNSCC samples, Cyclin D1 expression is significantly increased. Moreover, pRb is significantly upregulated in all datasets (Table 2 and Table 3). The Rb pathway is disrupted in most tumors. Deletion and inactivating mutations of pRb are restricted to very few specific cancer types. pRb absence in mouse epidermis is characterized by moderate hyperplasia and hyperkeratosis associated with increased proliferation and altered differentiation. However, no spontaneous tumor development was observed, even after a long latency, likely for the overlapping tumor suppressor roles of p107 and p130 [76]. Here, these proteins are slightly upregulated (Table 2 and Table 3). Interestingly, high pRb or combined pRb/CyclinD1 expression in HNSCCs are strong indicators for HPV-negative status [186], suggesting that the *p16^INK4a^* upregulation observed in these datasets may be independent of HPV infection. The pRb pathway effectors, E2F1 and E2F3, significantly increase in NMSCs. E2F2 displays a similar trend (Table 2 and Table 3), indicating a deregulation of this pathway. Indeed, amplification and increased expression of specific E2F members is frequent in NMSCs. Indeed, deregulation of E2F activity may be involved in the unregulated proliferation of skin tumor cells [187]. In HNSCC samples also E2F4 displays a significant increase (Table 3).

##### The *p16^INK4^* Epigenetic Modulators and Transcription Factors in Epithelial Tumors

Epigenetic modulators and transcription factors are dysregulated in cancers. However, poor data concerning modulation of epigenetic enzymes have been collected from skin tumors.

Analyzing these datasets, DNMT1 appears to be upregulated in cSCCs, BCCs, HNSCCs, and oSCCs. DNMT3A is mainly increased in BCCs whereas DNMT3B is upregulated in AKs, cSCCs, HNSCCs, and oSCCs (Table 2 and Table 3). An increase of three DNMT isoforms has been previously observed in BCCs where they contribute to hypermethylation and silencing of *p16^INK4a^*. This hypermethylation is accompanied by increased occupancy by MBD1, MeCP2, and HDACs [175]. Thus, other epigenetic and transcription factors might be responsible for *p16^INK4a^* upregulation in analyzed specimens.

Among PRC subunits, Ezh2, Bmi-1, and EED expression significantly increases in analyzed specimens (Table 2 and Table 3). Due to their role in cell cycle control and apoptosis, the PcG proteins Ezh2 and Bmi-1 are important epigenetic regulators enhancing skin cancer cell survival. Ezh2 expression increases from normal skin to AKs and further to cSCCs, suggesting a role in cSCC initiation and progression. Bmi-1 is highly expressed in both BCCs and cSCCs [88,89]. However, a strong Bmi-1 overexpression is more frequently observed in BCCs than in cSCCs and AKs [188]. In our analysis, Bmi-1 expression is significantly increased in BCCs and SCCs from one dataset (Table 2). Of note, Hedgehog pathway dysregulation upregulates Bmi-1, suggesting its critical role in BCC pathogenesis. Moreover, Bmi-1 overexpression in immortalized HaCaT cells causes malignant transformation both in vitro and in vivo [189]. Interestingly, a human cSCC cell subpopulation possesses SC-like features, has enriched expression of Ezh2, Bmi-1, and trimethylated histone H3, and display enhanced ability to drive tumor formation [190]. Specifically, Ezh2 is required for survival of these cells and tumor formation [191]. Of note, Bmi-1 overexpression restores Ezh2 levels in Epigallocatechin-3-gallate (i.e., the active agent in green tea)-treated cSCC cells, suggesting a feedback regulation to balance of PRC1 and PRC2 complex protein expression in cells [89]. Chemopreventive agents suppress proliferation by cyclin inhibition and/or induce apoptosis in cSCC cells. However, these effects are reversed by forced Bmi-1 expression [89,90]. Among the other PRC subunits, CBX4 expression is unaltered, whereas CBX7 is globally downregulated in this dataset analysis. The PRC2 ancillary protein Jarid2 significantly increases mainly in cSCCs. Of note, JMJD3 globally increases in NMSCs, whereas JDP2 is upregulated only in BCCs (Table 2 and Table 3).

MLL1 (KMT2A) expression is significantly increased in NMSCs (Table 2). Indeed, multiple mutations and copy number variations of MML1 have been found in SCCs [192].

The p63 activator Setd8 is upregulated only in SCCs. According to literature, p63 expression is significantly over-expressed in SCCs, BCCs, HNSCCs, and oSCCs (Table 2 and Table 3). Indeed, it is abundantly expressed in cSCCs and favors tumor initiation and progression [193]. For instance, p63 targets LSH to drive skin tumorigenesis [101]. Here, we find a strong and significant upregulation of LSH in NMSCs, observed also in HNSCCs and oSSCs (Table 2 and Table 3).

The HAT p300 is mainly upregulated in NMSCs whereas the HDACs are globally upregulated in all analyzed pathologies (Table 2 and Table 3). The p300 increase plays an important role in the development and progression of cSCC [194]. Moreover, high p300 expression correlates with aggressive features of cSCC, suggesting that p300 may be a promising biomarker to predict clinical outcomes [195]. Overexpression of the HDACs is frequent in oral cancer. Indeed, ΔNp63α/HDAC complex is necessary for tumor maintenance in SCCs as it serves as a direct repressor of the apoptotic transcriptional program [196].

Among the other p63 targets, BRG1 is significantly upregulated in NMSC specimens, differently from HNSCCs and oSCCs (Table 2 and Table 3). BRG1 is upregulated in esophageal SCCs and is necessary for matrix metalloproteinases expression [197]. Interestingly, SATB1 is slightly downregulated in all analyzed pathologies (Oncomine results). Of note, high SATB1 expression is predictive of poor prognosis in oral, laryngeal, and esophageal SCCs [198,199,200]. HBP1 is upregulated in cSCCs and BCCs differently from HNSCCs and oSCCs (Table 2 and Table 3). The differential expression of BRG1, Satb1, HBP1, and LSH might be responsible for *p16^INK4^* upregulation in cSCCs and BCCs.

B-myb is slightly upregulated in cSCCs and downregulated in the other specimens. CTFC expression is significantly increased in BCCs and cSCCs from one dataset (Table 2 and Table 3).

Ets transcription factors are upregulated in cSCCs, HNSCCs, and oSCCs from one dataset (Oncomine results). Ets2 is a major driver of SCCs. Ets2 binds to the super-enhancers and reprograms gene expression to induce inflammation and promote the tumor growth and development [201]. The *p16^INK4^* repressor Id1 is significantly upregulated in AKs, cSCCs, HNSCCs, and oSCCs whereas Id2 and Id3 increase only in AKs and HNSCCs (Table 2 and Table 3). In cSCCs, Id protein expression increase is observed in the majority of malignant poorly differentiated tumors compared to these well-differentiated ones. It might contribute to the escape of the relatively undifferentiated tumor cells in BCC from immune surveillance [110,202,203]. Id1 protects keratinocytes from apoptosis via the NF-kB/survivin and phosphoinositide 3-kinase/Akt signaling pathways [203].

The transcription factor AP-1 promotes the invasive growth and metastasis of various tumors such as SCCs [204]. AP-1 is composed of a mixture of homo- and hetero-dimers formed between JUN and FOS proteins that may have both opposite and overlapping functions in cellular proliferation and cell fate. Thus, we analyzed the expression of single proteins. JunB and JunD slightly increased in HNSCCs, AKs, and cSCCs from one dataset and downregulated in BCCs and SCCs of the other one. c-JUN is significantly increased in HNSCCs, AKs and cSCCs/oSCCs from one dataset (Table 2 and Table 3). JunB and JunD are negative regulators of the cell cycle, are not induced in a majority of SCC cells and inhibit the Ras-driven tumorigenesis. JunB directly induces *p16^INK4a^* expression and epidermal senescence. However, the dominant-negative JunB mutant promotes tumorigenesis [60,205]. Recently, JunB has been considered a key molecule in promoting cell invasion, migration, and distant metastasis in head and neck SCCs [206]. On the contrary, c-Jun is a positive regulator of cell cycle, is activated in SCCs and its coexpression with oncogenic Ras is sufficient to transform primary human epidermal cells into malignancy in a regenerated human skin grafting model [60,205].

c-Fos and FosB are downregulated in BCCs, cSCCs, and oSCC whereas they are strongly upregulated in HNSCCs. FRA1 and FRA2 are mainly upregulated in AKs and cSCCs (Table 2 and Table 3). Federico and colleagues do not find differential expression of c-Fos or FosB between tumors and normal tissues. On the contrary, a strong Fra-1 and Fra-2 expression is observed in those samples [207]. c-Fos is found in the specimens of patients with lymph node metastasis and is downregulated in poorly differentiated SCCs [208,209]. Moreover, c-Fos is a regulator of EMT and cancer stem cell reprogramming in head and neck SCC cells [210]. Fra-1 is highly expressed in SCCs, promotes tumor growth through the AKT pathway and enhances cell migration through JNK/c-Jun [211].

Clinical studies define ANRIL as a potential diagnostic and prognostic biomarkers in various cancers [212]. ANRIL can be considered an oncogene since it promotes cancer progression via increasing proliferation, reprogramming cell glucose metabolism and inducing CSCs [212,213]. Inhibition of ANRIL suppressed the cancer cell proliferation, migration, and invasion [213]. ANRIL has been found dysregulated in BCC [214]. However, in our analysis, ANRIL does not significantly vary in skin tumors and increases only in oSCCs from one dataset (Table 2 and Table 3).

In these specimens, *p16^INK4^* upregulation seems a compensatory mechanism to suppress tumor development by senescence. However, senescence of the non-epithelial component of developing skin tumours might enhance growth and invasion of the pre-malignant epidermal cells via other upregulated pathways, such as p63 and Ras. For instance, p63 is induced by oxidative stress. In the analyzed cutaneous tumors p63 targets (BRG1, Satb1, LSH) are globally upregulated. Ras is a transducer of extracellular mitogenic signals. Of note, in all analyzed tumors Ras targets (Cyclin D1, Ets1/2, HBP1, YB1, AP-1 proteins) are upregulated. Moreover, some of these proteins are also able to induce *p16^INK4^* expression to block uncontrolled growth.

As new data sets are deposited, it will be possible to analyze larger data sets and get more in-depth information.

## 7. Conclusions

*p16^INK4a^* is a potent inhibitor of the G1/S-phase transition of the cell cycle and exhibits a fundamental role in epidermal homeostasis, aging, and tumorigenesis.

Keratinocytes are equipped with several enzymes that shape the epigenetic signatures of different epidermal layers, allowing a dynamic and coordinated expression change of gene sets involved in self-renewal and differentiation. In the basal layer, epigenetic modifiers and transcription factors converge their activity to repress *p16^INK4a^* expression, protecting cells from senescence and preserving epidermal homeostasis and regeneration. Several stress stimuli may activate *p16^INK4a^* expression that orchestrates cell cycle exit and senescence response. Cellular senescence is a potent tumor suppressor mechanism, acting in coordination with the immune system to clear potentially malignant cells from the tissues. Thus, it may contribute to aging by depleting functional cells required to maintain tissue homeostasis. However, aging and cancer might be considered related endpoints of accumulating damages within self-renewing compartment. Indeed, age-associated changes in chromatin remodeling and gene expression are responsible for the deterioration of multiple cellular functions, including the senescence response. Accumulation of *p16^INK4a^* positive senescent cells in the aged tissue may be due to synergic contribution of the genomic instability, senescence-associated secretory phenotype dysregulation, and decline in the immune system function, and may contribute to worsening the senescence response efficacy in tumor suppression.

Unveiling the precise mechanisms regulating *p16^INK4a^* expression in human epidermal cell types may provide new targeted approaches to protect the epidermis from regenerative capacity decline and premature aging and new protocols for regenerative medicine. However, balancing the expression of *p16^INK4a^* and its regulators has to be carefully tuned to avoid the risk of malignant transformation, given that the response to oncogenic stimuli changes at advanced ages. Indeed, better understanding of *p16^INK4^* regulation in skin tumors may be relevant to standardizing new diagnostic or prognostic tools and in designing new cancer therapies.

## Figures and Tables

**Table 1:** Patient numerosity and Reference of each dataset investigated in the present study from Oncomine database (www.oncomine.org)

Tumor Type	Nindl Dataset [178]	Riker Dataset [179]	Ginos Dataset [180]	Cromer Dataset [181]	Peng Dataset [183]	Toruner Dataset [182]	TOT
Normal Tissue	6	4	13	4	22	4	53
Actinic (Solar) Keratosis	4	0	0	0	0	0	4
Skin Squamous Cell Carcinoma	5	11	0	0	0	0	16
Skin Basal Cell Carcinoma	0	15	0	0	0	0	15
Head and Neck Squamous Cell Carcinoma	0	0	41	34	0	0	75
Oral Cavity Squamous Cell Carcinoma	0	0	0	0	57	16	73

The table shows the tumors types, the datasets names, the patient numerosity, and the Reference of each dataset investigated in the present study, from Oncomine database (www.oncomine.org).

**Table 2:** Expression fold change of the genes investigated in the present study from Actinic (solar) Keratosis, Skin Squamous Cell Carcinoma, and Skin Basal Cell Carcinoma databases.

			Actinic (solar) Keratosis		Skin Squamous Cell Carcinoma		Skin Basal Cell Carcinoma		Skin Squamous Cell Carcinoma	
			Nindl Dataset				Riker Dataset			
			Cancer vs. Normal		Cancer vs. Normal		Cancer vs. Normal		Cancer vs. Normal	
n	Protein	Gene	Fold Change	*p* Value	Fold Change	*p* Value	Fold Change	*p* Value	Fold Change	*p* Value
1	**p16^INK4a^**	**CDKN2A**	**2.218**	**0.004**	**3.553**	**0.003**	**2.297**	**0.009**	**4.404**	**4.09 × 10^−4^**
*p16^INK4a^ Downstream Genes*										
2	**CDK4**	**CDK4**	−1.033	0.602	1.125	0.163	**1.976**	**5.34 × 10^−5^**	**1.713**	**8.07 × 10^−4^**
3	**Cyclin D1**	**CCND1**	2.393	0.053	1.513	0.226	1.006	0.492	1.002	0.496
4	**pRB**	**RB1**	**1.974**	**0.05**	1.349	0.17	**1.251**	**0.017**	**1.579**	**5.48 × 10^−4^**
5	**p107**	**RBL1**	n.a.	n.a.	n.a.	n.a.	1.307	0.284	1.9	0.11
6	**p130**	**RBL2**	1.632	0.152	1.676	0.163	1.367	0.141	**1.41**	**0.046**
7	**E2F1**	**E2F1**	**1.885**	**0.0037**	**2.071**	**0.028**	**1.604**	**0.011**	**1.749**	**0.004**
8	**E2F2**	**E2F2**	1.918	0.065	2.655	0.052	1.277	0.16	**1.769**	**0.028**
9	**E2F3**	**E2F3**	**1.759**	**0.006**	**1.78**	**0.006**	**3.28**	**2.41 × 10^−5^**	**2.617**	**3.08 × 10^−4^**
10	**E2F4**	**E2F4**	−1.072	0.638	1.114	0.089	−1.139	0.702	1.03	0.452
*Epigenetic Regulator Genes*										
11	**DNMT1**	**DNMT1**	1.473	0.068	**1.81**	**0.05**	**1.945**	**0.001**	**2.221**	**1.27 × 10^−4^**
12	**DNMT3A**	**DNMT3A**	−2.54	0.894	−3.426	0.969	**3.818**	**1.55 × 10^−8^**	**1.703**	**3.19 × 10^−4^**
13	**DNMT3B**	**DNMT3B**	**1.256**	**0.008**	**2.032**	**0.008**	1.079	0.258	**1.48**	**0.011**
14	**Bmi-1**	**BMI1**	−1.347	0.874	−1.536	0.939	**1.713**	**0.001**	**1.324**	**0.24**
15	**Ezh1**	**EZH1**	1.397	0.259	1.619	0.272	1.611	0.251	1.379	0.331
16	**Ezh2**	**EZH2**	1.671	0.059	**2.31**	**0.017**	**2.016**	**2.24 × 10^−4^**	**2.112**	**8.79 × 10^−5^**
17	**EED**	**EED**	−1.293	0.659	−1.043	0.567	1.231	0.074	**1.411**	**0.016**
18	**CBX4**	**CBX4**	−1.269	0.64	1.072	0.456	−1.082	0.762	1.006	0.486
19	**CBX7**	**CBX7**	−1.315	0.855	−1.993	0.997	−2.078	0.992	−2.923	0.999
20	**Jarid2**	**JARID2**	1.167	0.223	**1.409**	**0.021**	1.091	0.195	**1.382**	**0.006**
21	**JMJD3**	**JMJD3**	**1.568**	**0.018**	**1.562**	**0.017**	2.822	0.075	1.853	0.171
22	**JDP2**	**JDP2**	n.a.	n.a.	n.a.	n.a.	1.264	0.052	−1.147	0.821
23	**SETD8**	**KMT5A**	−1.282	0.629	1.411	0.102	1.06	0.346	**1.505**	**0.004**
24	**KMT2A**	**MLL**	**1.657**	**0.004**	**1.479**	**0.043**	**1.413**	**0.01**	1.184	0.076
25	**HAT p300**	**EP300**	**1.692**	**0.041**	**1.855**	**0.025**	**1.112**	**0.039**	**1.143**	**0.03**
26	**HDAC1**	**HDAC1**	1.066	0.414	**1.632**	**0.025**	**1.636**	**1.39 × 10^−5^**	**2.309**	**2.89 × 10^−7^**
27	**HDAC2**	**HDAC2**	−1.461	0.909	1.007	0.486	**2.223**	**1.19 × 10^−4^**	**1.682**	**0.001**
28	**p63**	**TP63**	1.452	0.234	**2.905**	**0.008**	**1.273**	**0.022**	**1.512**	**0.002**
29	**BRG1**	**SMARCA4**	**2.03**	**0.003**	**1.826**	**2.27 × 10^−4^**	**1.666**	**2.08 × 10^−6^**	**1.767**	**0.002**
30	**LSH**	**HELLS**	**4.504**	**0.039**	**8.371**	**0.016**	**5.292**	**3.02 × 10^−4^**	**1.948**	**0.003**
31	**Satb1**	**SATB1**	−1.125	0.853	−1.17	0.835	−1.342	0.865	1.25	0.813
32	**CTCF**	**CTCF**	−1.083	0.756	1.063	0.35	**1.54**	**1.72 × 10^−4^**	**1.277**	**0.006**
*Transcriptional Regulator Genes*										
33	**Ets1**	**ETS1**	1.31	0.759	1.446	0.13	1.139	0.329	**2.421**	**0.011**
34	**Ets2**	**ETS2**	1.247	0.102	**1.696**	**0.007**	−1.463	0.823	1.106	0.393
35	**ID1**	**ID1**	1.23	0.243	1.48	0.103	−1.824	0.995	**1.434**	**0.041**
36	**ID2**	**ID2**	**1.508**	**0.042**	−1.117	0.698	1.281	0.163	1.165	0.262
37	**ID3**	**ID3**	1.224	0.184	−1.512	0.92	−2.316	0.999	−1.137	0.687
38	**YB1**	**YXB1**	1.126	0.164	1.117	0.271	**1.314**	**0.005**	**1.48**	**0.001**
39	**SP1**	**SP1**	-1.699	0.769	1.847	0.082	1.048	0.223	1.112	0.317
40	**HBP1**	**PBRM1**	1.167	0.156	1.146	0.192	**1.513**	**5.96 × 10^−6^**	**1.984**	**0.031**
41	**b-myb**	**MYB**	−1.66	0.898	1.348	0.925	−1.14	0.593	1.592	0.204
42	**JUNB**	**JUNB**	1.107	0.292	1.375	0.078	−1.989	0.927	−1.09	0.586
43	**c-Jun**	**C-JUN**	**1.635**	**0.016**	**1.517**	**0.014**	1.055	0.455	1.04	0.468
44	**JunD**	**JUND**	1.16	0.121	1.058	0.401	−1.428	0.742	−1.558	0.8
45	**c-Fos**	**C-FOS**	−2.149	0.834	−3.068	0.937	−6.298	0.972	−1.95	0.814
46	**FosB**	**FOSB**	1.265	0.401	−6.929	0.96	−11.992	0.923	−8.365	0.896
47	**FRA1**	**FOSL1**	**2.66**	**0.048**	**2.937**	**0.021**	1.12	0.366	1.867	0.058
48	**FRA2**	**FOSL2**	**1.883**	**0.015**	**2.38**	**0.005**	1.339	0.065	**1.658**	**0.013**
49	**PPARalpha**	**PPARA**	−1.556	0.877	1.132	0.352	1.229	0.378	1.153	0.198
50	**ANRIL**	**CDKN2BAS**	n.a.	n.a.	n.a.	n.a.	−1.109	0.697	−1.152	0.744

The table shows the tumors types, dataset names, and complete list of genes investigated in the present study, from Oncomine database (www.oncomine.org). Statistically significant expression fold changes (*p* ≤ 0.05) are labeled: Fold change ≥ 2 (green); fold change < 2 (light green); n.a.: Not available data.

**Table 3:** Expression fold change of the genes investigated in the present study, from Head and Neck Squamous Cell Carcinoma and Oral Cavity Squamous Cell Carcinoma databases.

			Head and Neck Squamous Cell Carcinoma				Oral Cavity SquamousCell Carcinoma			
			Ginos Dataset		Cromer Dataset		Peng Dataset		Toruner dataset	
			Cancer vs. Normal		Cancer vs. Normal		Cancer vs. Normal		Cancer vs. Normal	
n	Protein	Gene	Fold Change	*p* Value	Fold Change	*p* Value	Fold Change	*p* Value	Fold Change	*p* Value
1	**p16INK4a**	**CDKN2A**	**2.297**	**6.27 × 10^−5^**	−1.415	0.712	**1.684**	**1.21 × 10^−7^**	**1.372**	**0.047**
*p16^INK4a^ Downstream Genes*										
2	**CDK4**	**CDK4**	**1.842**	**8.84 × 10^−12^**	**2.63**	**8.00 × 10^−6^**	**1.498**	**3.76E−08**	**2.13**	**6.31 × 10^−4^**
3	**Cyclin D1**	**CCND1**	**1.465**	**0.023**	**2.031**	**5.97 × 10^−5^**	−1.112	0.907	1.201	0.164
4	**pRB**	**RB1**	**1.372**	**8.92 × 10^−4^**	1.165	0.429	**1.2**	**0.018**	**1.579**	**0.005**
5	**p107**	**RBL1**	n.a.	n.a.	−2.032	0.97	**1.334**	**4.47 × 10^−4^**	n.a.	n.a.
6	**p130**	**RBL2**	1.081	0.172	−1.449	0.774	−1.228	0.997	**1.141**	**0.046**
7	**E2F1**	**E2F1**	**1.347**	**0.046**	**1.754**	**1.00 × 10^−6^**	1.032	0.313	**1.083**	**0.05**
8	**E2F2**	**E2F2**	−1.329	0.856	−1.147	0.813	−1.424	1	1.044	0.172
9	**E2F3**	**E2F3**	**2.945**	**7.20 × 10^−8^**	1.948	0.159	**1.099**	**4.19 × 10^−15^**	1.231	0.284
10	**E2F4**	**E2F4**	**1.131**	**0.025**	**1.462**	**0.014**	**1.415**	**1.28 × 10^−11^**	−1.178	1.00
*Epigenetic Regulator Genes*										
11	**DNMT1**	**DNMT1**	**1.706**	**2.00 × 10^−7^**	1.581	0.124	**1.499**	**1.87 × 10^−15^**	**1.457**	**2.00 × 10^−3^**
12	**DNMT3A**	**DNMT3A**	−1.046	0.584	n.a.	n.a.	1.01	0.37	−1.038	0.644
13	**DNMT3B**	**DNMT3B**	**1.37**	**5.19 × 10^−4^**	n.a.	n.a.	**1.512**	**3.82 × 10^−8^**	**1.296**	**0.009**
14	**Bmi-1**	**BMI1**	**1.818**	**3.23 × 10^−7^**	**1.627**	**5.16 × 10^−5^**	−1.046	0.689	**1.708**	**2.78 × 10^−7^**
15	**Ezh1**	**EZH1**	**1.238**	**0.011**	1.383	0.083	**1.131**	**6.00 × 10^−2^**	**1.259**	**0.03**
16	**Ezh2**	**EZH2**	−1.325	0.88	−1.151	0.833	−1.401	1.000	1.021	0.4
17	**EED**	**EED**	**1.495**	**3.89 × 10^−4^**	1.118	0.286	**1.349**	**3.12 × 10^−7^**	**1.112**	**0.013**
18	**CBX4**	**CBX4**	−1.133	0.750	−1.132	0.613	−1.183	0.999	−1.04	0.817
19	**CBX7**	**CBX7**	−1.429	0.992	n.a.	n.a.	−1.311	1	−1.24	1
20	**Jarid2**	**JARID2**	**1.123**	**0.045**	**1.17**	**0.039**	**1.2**	**0.002**	1.365	0. 948
21	**JMJD3**	**JMJD3**	1.039	0.292	1.127	0.116	1.088	0.174	−1.229	0.924
22	**JDP2**	**JDP2**	n.a.	n.a.	n.a.	n.a.	−1.214	1	n.a.	n.a.
23	**SETD8**	**KMT5A**	−1.375	0.999	n.a.	n.a.	1.058	0.214	**1.097**	**2.19 × 10^−4^**
24	**KMT2A**	**MLL**	−1.185	0.974	−1.252	0.901	**1.179**	**0.005**	1.11	0.215
25	**HAT p300**	**EP300**	1.054	0.327	−1.196	0.737	**1.141**	**0.006**	−1.146	0.904
26	**HDAC**	**HDAC1**	−1.075	0.83	1.361	0.054	**1.174**	**0.016**	**1.883**	**1.42 × 10^−4^**
27	**HDAC**	**HDAC2**	**1.613**	**1.41 × 10^−4^**	1.181	0.241	1.066	0.151	1.265	0.029
28	**p63**	**TP63**	**1.491**	**0.001**	**1.717**	**0.019**	**1.238**	**2.68 × 10^−4^**	**1.12**	**0.021**
29	**BRG1**	**SMARCA4**	1.047	0.309	−1.543	0.960	1.001	0.416	**1.175**	**9.28 × 10^−4^**
30	**LSH**	**HELLS**	**2.463**	**0.013**	n.a.	n.a.	1.617	6.24 × 10^−6^	**1.445**	**0.004**
31	**Satb1**	**SATB1**	1.025	0.396	−2.039	0.92	−1.496	0.997	−1.685	1
32	**CTCF**	**CTCF**	1.003	0.478	1.292	0.018	−1.114	0.998	1.29	0.097
*Transcriptional Regulator Genes*										
33	**Ets1**	**ETS1**	**1.694**	**0.001**	**2.076**	**0.016**	**2.111**	**2.97 × 10^−10^**	−1.114	1
34	**Ets2**	**ETS2**	**1.78**	**1.76 × 10^−6^**	**1.761**	**0.012**	−1.102	0.908	−1.218	0.618
35	**ID1**	**ID1**	**1.356**	**0.015**	1.375	0.278	**1.21**	**0.025**	1.242	0.317
36	**ID2**	**ID2**	**1.765**	**1.79 × 10^−4^**	2.022	0.258	1.066	0.193	1.038	0.362
37	**ID3**	**ID3**	**3.023**	**2.14 × 10^−4^**	n.a.	n.a.	1.072	0.208	1.043	0.447
38	**YB1**	**YXB1**	**1.421**	**0.001**	**1.644**	**6.26 × 10^−4^**	−1.362	0.998	**1.637**	**0.001**
39	**SP1**	**SP1**	1.292	0.95	−1.382	0.717	−1.232	1	1.008	0.372
40	**HBP1**	**PBRM1**	−1.131	0.716	n.a.	n.a.	−1.303	1.00	1.039	0.137
41	**b-myb**	**MYB**	1.003	0.495	1.414	0.13	−1.842	1	1.134	0.229
42	**JUNB**	**JUNB**	**1.38**	**0.006**	1.252	0.093	1.003	0.485	−1.029	0.552
43	**c-Jun**	**C-JUN**	**1.549**	**1.13 × 10^−5^**	1.18	0.083	**1.318**	**0.008**	−1.093	0.508
44	**JunD**	**JUND**	−1.048	0.549	**1.176**	**0.05**	−1.227	0.999	1.045	0.086
45	**c-Fos**	**C-FOS**	**13.503**	**2.88 × 10^−5^**	1.481	0.212	−1.415	0.956	1.225	0.365
46	**FosB**	**FOSB**	**3.016**	**0.005**	**1.385**	**0.021**	−1.042	0.527	−1.881	1
47	**FRA1**	**FOSL1**	1.663	0.076	−1.194	0.922	**1.2**	**0.045**	−3.719	0.946
48	**FRA2**	**FOSL2**	−1.355	0.997	1.13	0.205	−2.032	1	−1.149	0.909
49	**PPARalpha**	**PPARA**	−1.043	0.406	−1.134	0.585	−1.079	0.984	1.021	0.104
50	**ANRIL**	**CDKN2BAS**	n.a.	n.a.	n.a.	n.a.	**1.802**	**8.85 × 10^−12^**	n.a.	n.a.

The table shows the tumors types, dataset names, and complete list of genes investigated in the present study, from Oncomine database (www.oncomine.org). Statistically significant expression fold changes (*p* ≤ 0.05) are labeled: fold change ≥ 2 (green); fold change < 2 (light green); n.a.: not available data.
